# Breastfeeding patterns are associated with human milk microbiome composition: The Mother-Infant Microbiomes, Behavior, and Ecology Study (MIMBES)

**DOI:** 10.1371/journal.pone.0287839

**Published:** 2023-08-09

**Authors:** Elizabeth A. Holdsworth, Janet E. Williams, Ryan M. Pace, Avery A. Lane, Maria Gartstein, Mark A. McGuire, Michelle K. McGuire, Courtney L. Meehan

**Affiliations:** 1 Department of Anthropology, Washington State University, Pullman, Washington, United States of America; 2 Department of Animal, Veterinary and Food Sciences, University of Idaho, Moscow, Idaho, United States of America; 3 Margaret Ritchie School of Family and Consumer Sciences, University of Idaho, Moscow, Idaho, United States of America; 4 Department of Psychology, Washington State University, Pullman, Washington, United States of America; Emory University School of Public Health, UNITED STATES

## Abstract

The human milk microbiome (HMM) is hypothesized to be seeded by multiple factors, including the infant oral microbiome during breastfeeding. However, it is not known whether breastfeeding patterns (e.g., frequency or total time) impact the composition of the HMM. As part of the Mother-Infant Microbiomes, Behavior, and Ecology Study (MIMBES), we analyzed data from naturalistic observations of 46 mother-infant dyads living in the US Pacific Northwest and analyzed milk produced by the mothers for its bacterial diversity and composition. DNA was extracted from milk and the V1-V3 region of the 16S rRNA gene was amplified and sequenced. We hypothesized that number of breastfeeding bouts (breastfeeding sessions separated by >30 seconds) and total time breastfeeding would be associated with HMM α-diversity (richness, diversity, or evenness) and differential abundance of HMM bacterial genera. Multiple linear regression was used to examine associations between HMM α-diversity and the number of breastfeeding bouts or total time breastfeeding and selected covariates (infant age, maternal work outside the home, frequency of allomother physical contact with the infant, non-household caregiving network). HMM richness was inversely associated with number of breastfeeding bouts and frequency of allomother physical contact, but not total time breastfeeding. Infants’ non-household caregiving network was positively associated with HMM evenness. In two ANCOM-BC analyses, abundances of 5 of the 35 most abundant genera were differentially associated with frequency of breastfeeding bouts (*Bifidobacterium*, *Micrococcus*, *Pedobacter*, *Acidocella*, *Achromobacter*); 5 genera (*Bifidobacterium*, *Agreia*, *Pedobacter*, *Rugamonas*, *Stenotrophomonas*) were associated with total time breastfeeding. These results indicate that breastfeeding patterns and infant caregiving ecology may play a role in influencing HMM composition. Future research is needed to identify whether these relationships are consistent in other populations and if they are associated with variation in the infant’s gastrointestinal (including oral) microbiome.

## Introduction

The human milk microbiome (HMM) represents a major source of microbial exposure for the breastfed infant and may be an important driver of infant growth and development. For example, some have hypothesized that the HMM beneficially impacts infant gene expression and immune development [1,2]. This may be, at least in part, due to putative impacts of the HMM on the recipient infant’s gastrointestinal (GI) microbiome, which has been associated with infant development [3]. In addition, the infant GI microbiome is itself a predictor of later-life health and disease outcomes including asthma, allergy, obesity, and neuropsychiatric disorders [3,4]. As such, the HMM is hypothesized to represent an important pathway for intergenerational influences on early life origins of health and disease.

There are presumably many factors that influence variation in the HMM, although little is known about their mechanisms, in large part because studying these relationships has been complicated by differences in populations, sample collection and analysis protocols, and data analytic methods including selection of covariates [4,5]. Nonetheless, the HMM is hypothesized to be seeded by multiple sources including the maternal GI, breast skin, and areola microbiomes [6] and possibly the infant oral microbiome [7], meaning that individual and environmental factors likely contribute to HMM variation. Furthermore, diversity of the HMM has been found to be related to maternal characteristics and behaviors such as geographic location [8,9], time postpartum [10], body mass index (BMI), delivery mode including elective vs. nonelective cesarean deliveries [11], mastitis [12], nutrition [13], and social and caregiving patterns [14].

After birth, the HMM is thought to change over the course of lactation [10,15] although data are not consistent in this regard [13]. These changes, if they exist, may be a result of increasing postnatal bacterial translocation from the maternal GI tract [5,16], as well as external influences such as the infant oral microbiome seeding the mammary gland through nursing. Previous research has suggested retrograde flow of milk and saliva from the infant oral cavity into the mammary gland may occur [7], resulting in a potential influence of breastfeeding practices on HMM composition [17,18]. Supporting this is the finding that HMM composition is similar to infant oral microbiome composition in mother-infant dyads (including more similar than HMM composition to infant fecal microbiome), and variation in HMM composition and variation in infant oral microbiome composition are associated with each other [19]. The possible seeding of the HMM by the infant oral microbiome is further complicated by the findings that the infant oral microbiome can be influenced by external environmental and infant-specific factors including diet and transmission from caregivers and peers [20–23]. These findings mean that infant bacterial exposures, exclusive of the mother’s microbiome and household co-habitants [24] (e.g., how frequently infants are in physical contact with other caregivers and the range of individuals outside of the household who have physical contact with the infant), may also contribute to the infant oral microbiome and, in turn, the HMM. As such, while maternal environmental and individual factors are associated with variation in HMM composition and the infant oral microbiome, so too are infant environmental and individual factors, comprising a potentially important and dynamic mother-infant-environment influence on the HMM [14].

This dynamic interplay between mothers and infants has been proposed as the mother-infant nexus framework, which considers the dyad as a biocultural synthesis: a single, entwined unit, rather than discrete interacting entities [25]. Understanding the contribution of the mother-infant nexus to human biological variation is critical to understanding the evolution of humans and mother-infant health and caregiving strategies. Breastfeeding patterns are a mother-infant nexus point; it is one of many dynamic, interactive components of the mother-infant relationship. Within this framework, we hypothesize that breastfeeding patterns contribute to HMM variation when accounting for the unique microbial inputs from mothers and infants separately.

Breastfeeding practices vary widely across and within populations and over time postpartum [26], and breastfeeding is a biosocial activity. Breastfeeding delivers nutritive and non-nutritive milk-borne components to the infant and, as an activity, its structure and patterning are culturally, behaviorally, and ecologically informed [27]. The temporal patterning of breastfeeding is facilitated or constrained by many factors including the environment, maternal-infant health (including nutrition), cultural practices, time postpartum, parental ideologies of caregiving and infant agency (e.g., whether the act is viewed as mother or infant directed), maternal work patterns, and the availability of allomaternal caregivers [26–28]. For instance, examples abound in the literature on hunter-gatherer breastfeeding patterns, which are characterized as on-demand (multiple bouts per hour and infant-directed) [26,29–31]. That pattern is embedded in environments and cultures that enable consistent maternal-infant skin-to-skin contact throughout the day and night, indulgent and responsive caregiving, and parental ideologies that value infant autonomy and view infants as the active agent in the breastfeeding process [27,32]. In contrast, Hewlett notes that western women (those from WEIRD—Western, educated, industrialized, rich and democratic—societies [33]) more frequently practice a form of pulse feeding, characterized by longer breastfeeding episodes and prolonged time between episodes [26]. Within this latter population, however, substantial variation in breastfeeding behaviors exist.

Despite the wide variety of sociocultural influences on breastfeeding patterns, however, few studies have explored the relationship between breastfeeding practices and the HMM. In one study of non-exclusively breastfed infants and mothers that used non-aseptic milk collection methods, frequency of direct breastfeeding was associated with HMM beta-diversity and with the reduced relative abundance of *Corynebacterium* and *Staphylococcus* [34]. While this study provides compelling initial evidence for a relationship between breastfeeding patterns and HMM variation, it is unknown whether the same patterns will be present in analysis of an exclusively breastfeeding sample where the breast was washed prior to milk collection. Additionally, no studies have used direct behavioral observation of breastfeeding patterns, which provide data on time spent breastfeeding and the structure of the activity, nor accounted for the independent association between the infant’s caregiving environment and the HMM [14].

We hypothesize that variation in breastfeeding practices, including total time breastfeeding and frequency of breastfeeding bouts, are associated with HMM α-diversity and differential abundance of some bacterial genera, accounting for bacterial exposure through environmental vectors not shared by the mother-infant dyad.

## Materials and methods

### Study design and participants

Fifty-one mother-infant dyads residing in rural eastern Washington and northeast Idaho were enrolled in the Mother-Infant Microbiomes, Behavior, and Ecology Study (MIMBES). All women were exclusively breastfeeding or pumping at least 5 times per day (if pumping, also exclusively feeding their own milk to their infants) to ensure adequate milk production, ≥18 years of age, 3 weeks to 5.5 months postpartum, and reported themselves and their infants as healthy. Potential participants were excluded if they self-reported any indications of breast infection in the previous 7 days (i.e., fever, red streaks on breast, hard red portions on breast, any abnormal breast pain, discomfort, or lumps), infant or maternal signs of acute illness in the previous 7 days (i.e., fever, diarrhea, vomiting, severe cough, or rapid breathing in infants), or any antibiotics consumed by or administered to the mother or infant in the past 30 days. Mothers completed surveys at the start of the study, participated in behavioral observations of the infant, and collected/submitted a milk sample for microbiome analysis. This analysis includes only those women whose milk samples had sequencing read counts >200, resulting in 46 mother-infant dyads. Written informed consent to participate was provided by women, and assent was provided for their infants. This study was approved by the Institutional Review Board of Washington State University (#15852).

### Metadata

Study personnel delivered a questionnaire to participants and recorded their responses. Data collected included maternal age in years, participant-supplied term of ethnic identity, their infants’ age in days at first data collection point (transformed to months for this analysis), and maternal work activities (paid or volunteer, part-time or full-time) outside the home. Self-identified ethnicity is reported here to provide sociocultural context for the sample population and should not be used as a proxy for biological or genetic differences.

### Infant observational data

Naturalistic observations were conducted on each of the participating infants. Mothers and infants continued their daily activities, both within and outside the home during the observations. These infant focal observations have successfully documented infant behavior and care across multiple populations and countries, including in the United States [27,35–38]. Observations occurred over the course of 3 days from 0700 to 1900 h and were divided into 4-hour segments (0700–1100, 1100–1500, and 1500–1900 h). Observations of select behaviors were recorded every 30 seconds. The observer took a 15-minute break after 45 minutes of observation to avoid observer fatigue. The resultant data documented 9 out of 12 hours (due to the 15-minute breaks) of infant behavior, instances of breastfeeding, and the interactions of infants with their caregivers. All individuals interacting with the focal infant were provided a unique identifier, enabling analysis of different categories of caregivers’ interactions with the infant. Caregivers were placed into 11 possible categories: mother, father, grandmother, sister, brother, juvenile girl, juvenile boy, adult woman, adult man, elderly woman, and elderly man. Juvenile, adult, and elderly categories could include family relatives (e.g., cousins, aunts and uncles, or great-aunts and great-uncles) or non-family. Categories were exclusive; individuals could not occupy more than one category.

The infant’s caregiving ecology was characterized by two measures derived from observational data: 1) allomother (non-maternal) physical contact frequency and 2) richness of the non-household caregiving network. These measures were selected to characterize potential infant microbial exposures exclusive from those shared with their mother (i.e., allomother physical contact frequency), and infant exposure to microbial sources from those specifically outside the home, as there is similarity between household cohabitants’ microbiomes [24]. Frequency of allomother physical contact was defined as any physical contact (touching or holding) of the infant by anyone who was not the mother in each observable interval (30 seconds). We measured the infant’s non-household caregiving network by calculating a caregiver richness measure (number of categories of non-household individuals who had observed physical contact with the infant, i.e., grandmothers, adult women, adult men, elderly women, elderly men, non-sibling girls, and non-sibling boys).

Total time breastfeeding was calculated as the total number of minutes across the 9 hours of observation that the infant was recorded as breastfeeding. Breastfeeding bouts were defined as episodes of breastfeeding separated by >30 seconds. One participant had a substantially greater amount of total time breastfeeding (313 minutes). To minimize the influence of this extreme outlier on the bivariate and multivariable analyses, this value was Winsorized to one minute above the next highest value, resulting in a value of 179 minutes.

For ease of interpretation, allomother physical contact frequency, total time breastfeeding, and breastfeeding bouts were transformed to z-scores. Variables had very different ranges before transformation (e.g., maternal work outside the home was dichotomous 0–1, while allomother frequency of physical contact ranged from 0–453). Transforming wider-range variables to z-scores facilitates comparisons to variables with smaller ranges in the regression models. Total time breastfeeding was transformed to z-scores after adjusting the outlier as described previously.

### Milk sample collection

Each participant provided a milk sample via full breast expression to capture both hind- and foremilk. Milk samples were collected between 0700–1100 h in the laboratory after having not nursed for at least 2 hours on the “study breast” after the period of behavioral observations described above. After cleaning the breast with a castile soap wipe, milk was collected using a Medela electric breast pump and single-use sterile milk collection kit. Samples were swirled and immediately aliquoted into vials and transferred to a freezer (-20°C). Following completion of the study, samples were transferred to the University of Idaho where they were stored at -20°C until sample analysis.

### Extraction of DNA from milk and bacterial DNA amplification

DNA was extracted from 1 mL of each milk sample following a modified protocol using enzymatic lysis and bead-beating and then the QIAamp DNA Mini Kit (Qiagen, Germantown, MD) as described previously [9]. DNA was eluted with 50 μl nuclease free water and stored at -80°C until further processing. The V1-V3 hypervariable region of the 16S rRNA gene was amplified using a dual-barcoded two-step 30-cycle polymerase chain reaction (PCR) as described previously [9].

### DNA quality, quantification, pooling, and sequencing

PCR amplicons were cleaned and size selected using paramagnetic beads from the HighPrep PCR Clean-up System (MagBio Genomics Inc., Gaithersburg, MD) following manufacturer’s instructions. Cleaned amplicons were quantified using the Accuclear Ultra High Sensitivity dsDNA Quantitation Kit (Biotium, Fremont, CA) and pooled to contain 50 ng DNA from each sample. Amplicon pools were then cleaned using paramagnetic beads, quality checked on a Fragment Analyzer (Advanced Analytical Technologies, Ankeny, IA, USA), and quantified using the KAPA Biosciences Illumina library quantification kit and Applied Biosystems StepOne Plus real-time PCR system. Pools were sequenced using an Illumina MiSeq (San Diego, CA) v3 paired-end 300-bp protocol for 600 cycles at the University of Idaho Genomics and Bioinformatics Resources Core.

### Sequence analysis

Sequencing reads were demultiplexed using dbcAmplicons (https://github.com/msettles/dbcAmplicons) and processed using DADA2 and decontam packages in R [39,40] as described by Pace and colleagues [41]. One sample was removed from the dataset through the decontam process. Four samples with <200 reads were removed from the dataset based on evaluation of ASV rarefaction curves.

### Statistical analyses

#### Calculation of α-diversity indices

All statistical analyses were conducted in R version 4.2.2 [42]. Milk microbial data were subsampled to an even sampling depth of 200 reads prior to calculating diversity indices using the rarefy function in the R vegan package [43]. α-diversity indices (observed richness and Shannon diversity) were calculated using the “estimate_richness” function in the R phyloseq package [44]. Richness is a count of the total distinct ASVs observed in a sample. Shannon diversity is a composite index of richness and evenness that is more sensitive to abundances of rare ASVs than dominant ASVs. We selected Shannon diversity to characterize HMM diversity as we anticipated that variation across this population would be more apparent in the abundance of rare ASVs. Shannon evenness was calculated as Shannon diversity/ln(observed richness).

#### Descriptive and bivariate statistics

Univariate statistics on all covariates were conducted. Differences of covariate values between groups of total breastfeeding time and breastfeeding bouts were tested for significance with ANOVAs for continuous variables, chi-squared tests for the categorical variable of maternal work outside the home, and Kruskal-Wallis rank sum tests for the discrete variable of infant non-household caregiving network. Bivariate statistics were evaluated using Pearson correlation coefficients. Significance was declared at p<0.05.

#### Multiple linear regression models

Six multiple linear regression models were created for each dependent variable of the α-diversity indices (stats::lm function [42]). These models included the main predictor of interest (number of breastfeeding bouts in the observed period z-scores or total time breastfeeding z-scores), infant age (months), and any maternal work outside the home. Separate models were run for the main predictor variables of breastfeeding bouts and total time breastfeeding given that these are not totally independent variables from each other. Models predicting Shannon diversity and Shannon evenness also included the covariate of non-household caregiving network. Models predicting richness included the covariate of z-scores of allomother physical contact frequency. These covariates were chosen in these models to best characterize the infant’s microbiome-relevant ecology exclusive of maternal effects, while also improving model fit as assessed by model adjusted R-squared and p-values. We explored other regression models using the same measures of infant caregiving ecology across the models; these models performed poorly and resulted in worse model fit (Tables A-C in S1 Table). Significance was declared at p<0.05.

#### Analysis of Composition of Microbiomes with Bias Correction (ANCOM-BC)

To evaluate whether abundance of specific genera differed by frequency of breastfeeding or total time breastfeeding, we conducted an ANCOM-BC on the unrarefied read count data of samples with >200 reads using the ANCOM-BC package in R, version 1.6.1 [45]. ANCOM-BC estimates differences in absolute abundance of taxa while correcting for the bias of unknown sampling fractions and allows for regression modeling to account for relevant covariates, as well as correct for multiple testing. Breastfeeding bouts and total breastfeeding time were grouped into low (<-1 z-scores), medium (-1 to 1 z-scores), and high (>1 z-scores), with medium as the reference group, as ANCOM-BC conducts global statistical tests across groups, rather than continuous variables. These groups were selected to compare differences between the extremes of breastfeeding bouts and total time breastfeeding, while maintaining adequate sample sizes for each group. Alpha was set to 0.05. P-values were adjusted for multiple testing using the Benjamini-Hochberg procedure. Significance was determined as q<0.05. Given the small sample size, this analysis also used a conservative variance estimate of the test statistic. Taxa with a proportion of zeroes greater than 0.85 were excluded from the analysis, and analysis was set to detect structural zeroes across groups. Covariates included infant age (months), any current maternal work outside the home, and allomother physical contact frequency.

## Results

### Participant characteristics

Participants (n = 46) were on average 29 years of age (Table 1). Most participants self-identified as White, Caucasian, or European American (n = 39, 84.8%), while other participants self-identified as Asian or Asian American (n = 3, 6.5%), other (n = 3, 6.5%), and Hispanic or Latino (n = 1, 2.2%). Mean infant age was 2.6 months, ranging from 29 to 176 days.

**Table 1:** Descriptive statistics, caregiving characteristics, breastfeeding, and HMM α-diversity metrics of participants, related to total time breastfeeding and breastfeeding bouts groups.

		Total Time Breastfeeding Groups			Breastfeeding Bouts Groups		
	Total sample (n = 46)	Low (<1 z-score) group (n = 10)	Medium [–1,1] z-score group (n = 28)	High (>1 z-score) group (n = 8)	Low (<1 z-score) group (n = 10)	Medium [–1,1] z-score group (n = 30)	High (>1 z-score) group (n = 6)
Maternal age (years)	29.1 ± 3.8	28.5 ± 4.3	29.4 ± 3.7	28.6 ± 3.8	29.5 ± 3.9	28.9 ± 4.0	29.5 ± 3.2
Infant age (months)	2.56 ± 1.41	3.37 ± 1.73	2.42 ± 1.14	2.03 ± 1.60	2.87 ± 1.21	2.59 ± 1.56	1.86 ± 0.68
Mother currently working at least part-time outside home	22 (47.8%)	4 (40.0%)	12 (42.9%)	6 (75.0%)	6 (60.0%)	13 (43.3%)	3 (50.0%)
Non-household caregiving network (count of categories)	0.5 ± 0.8	0.5 ± 0.7	0.7 ± 0.9	0.0 ± 0.0	0.5 ± 1.0	0.6 ± 0.9	0.2 ± 0.4
Allomother physical contact frequency (# of observations)^1^	70.7 ± 85.2**	97.7 ± 73.1	71.3 ± 96.4	35.1 ± 38.4	143.3 ± 142.6	50.7 ± 48.7	50.2 ± 39.6
Total time breastfeeding over observation period (minutes)	72.6 ± 38.5**	25.6 ± 6.8	71.3 ± 19.8	136.1 ± 13.8	36.3 ± 22.5	76.4 ± 31.6	114.4 ± 42.8
Breastfeeding bouts over observation period (#)	11.6 ± 4.5*	7.8 ± 4.2	11.9 ± 3.9	15.6 ± 3.0	5.1 ± 1.1	12.4 ± 2.2	18.7 ± 1.4
Richness of HMM	26.3 ± 5.8	27.0 ± 4.0	26.3 ± 5.5	25.9 ± 8.7	27.0 ± 6.6	26.8 ± 5.5	23.0 ± 5.4
Shannon diversity of HMM	2.45 ± 0.44	2.57 ± 0.30	2.47 ± 0.41	2.24 ± 0.65	2.45 ± 0.40	2.51 ± 0.44	2.16 ± 0.46
Shannon evenness of HMM	0.75 ± 0.10	0.78 ± 0.06	0.76 ± 0.09	0.69 ± 0.15	0.75 ± 0.08	0.76 ± 0.11	0.69 ± 0.11

Statistics of total time breastfeeding and total time breastfeeding groups are calculated after adjusting an outlier. Tests of differences across groups were ANOVAs for continuous variables, chi-squared tests for categorical variable of maternal work outside the home, and Kruskal-Wallis rank sum tests for discrete variable of infant non-household caregiving network. Cell entries are mean ± SD or n (%).

^1^ Allomother physical contact refers to the number of observations in which an allomother was in physical contact with the infant. Observation units were in 30 second intervals.

* Significantly different between <-1, [–1,1] and >1 z-scores of total time breastfeeding.

** Significantly different between <-1, [–1,1], and >1 z-scores of breastfeeding bouts.

### Breastfeeding structure

The mean number of breastfeeding bouts over 9 observed hours was 11.6, meaning infants had on average more than one bout per hour (Table 1). Total time breastfeeding ranged from 17 to 179 minutes (after adjusting an outlier as described previously), and the mean was 73.9 minutes. Breastfeeding bouts and total time breastfeeding showed variation in patterns across participants (S1 Fig), with some participants demonstrating less overall time breastfeeding, but with many bouts of breastfeeding throughout the day, and other participants demonstrating more overall time breastfeeding spread over a fewer number of bouts. However, time breastfeeding and number of breastfeeding bouts were positively correlated (r = 0.60, p<0.001).

### Caregiving context

Nearly half the women (48%) in this analytic sample worked or volunteered outside the home at least part time. There was a wide range in the frequency (0–453 instances) of allomother physical contact (how many times a person who was not the infant’s mother had any physical contact with the infant) over the 9 hours of observation. Allomothers who had physical contact with the infant included fathers (0–153 instances of physical contact), adult women (0–191 instances), adult men (0–41 instances), grandmothers (0–419 instances), sisters (0–75 instances), brothers (0–37 instances), elderly women (0–98 instances), girls (0–53 instances), and boys (0–10 instances). The richness of the non-household caregiving network ranged from 0 to 3, with an average of 0.52 categories of non-household members who had any observed physical contact with the infant. Most non-household caregivers were in the category of adult women (23.9%), followed by boys (8.7%), grandmothers (6.5%), girls (6.5%), adult men (4.3%), and elderly women (2.2%). No non-household elderly men had any physical contact with the infants.

### Differences in population characteristics by breastfeeding patterns

Some population characteristics significantly differed across breastfeeding bouts groups (defined as low <-1, medium = [–1,1], and high >1 z-scores of breastfeeding bouts) including mean total time breastfeeding (p<0.001) and allomother physical contact frequency (p = 0.007). Mean allomother physical contact frequency was significantly higher in the low breastfeeding bouts group compared to the medium (p<0.01) and high breastfeeding bouts groups (p = 0.03, S2A Fig). Mean number of breastfeeding bouts also significantly differed (p<0.001) across total time breastfeeding groups (defined as low <-1, medium = [–1,1], and high >1 z-scores of total time breastfeeding). Mean richness of the infant’s non-household caregiving network was not significantly different (p = 0.09) across total time breastfeeding groups. Infants’ non-household caregiving network means were significantly smaller in the highest total time breastfeeding group than the medium total time breastfeeding group in pairwise t-tests (p = 0.04, S2A Fig).

Mean infant age was not significantly different (p = 0.09) across total time breastfeeding groups, but there were on average significantly younger infants in the highest total time breastfeeding group compared to those in the lowest total time breastfeeding group in pairwise t-tests (p = 0.05). Boxplots showing the distribution of data across these groups and significant pairwise t-tests between groups are available in S2A Fig.

### Description of human milk microbiome

After quality control, sequence data of the V1-V3 region of the bacterial rRNA gene were available for milk produced by 46 mothers. Counts of bacterial V1-V3 rRNA reads ranged from 203–1822, with a mean ± s.d. of 1248 ± 882. Mean richness was 26.3 (median = 26.0); mean Shannon diversity was 2.45 (median = 2.52); and mean Shannon evenness was 0.75 (median = 0.76; Table 1).

**Fig 1:**
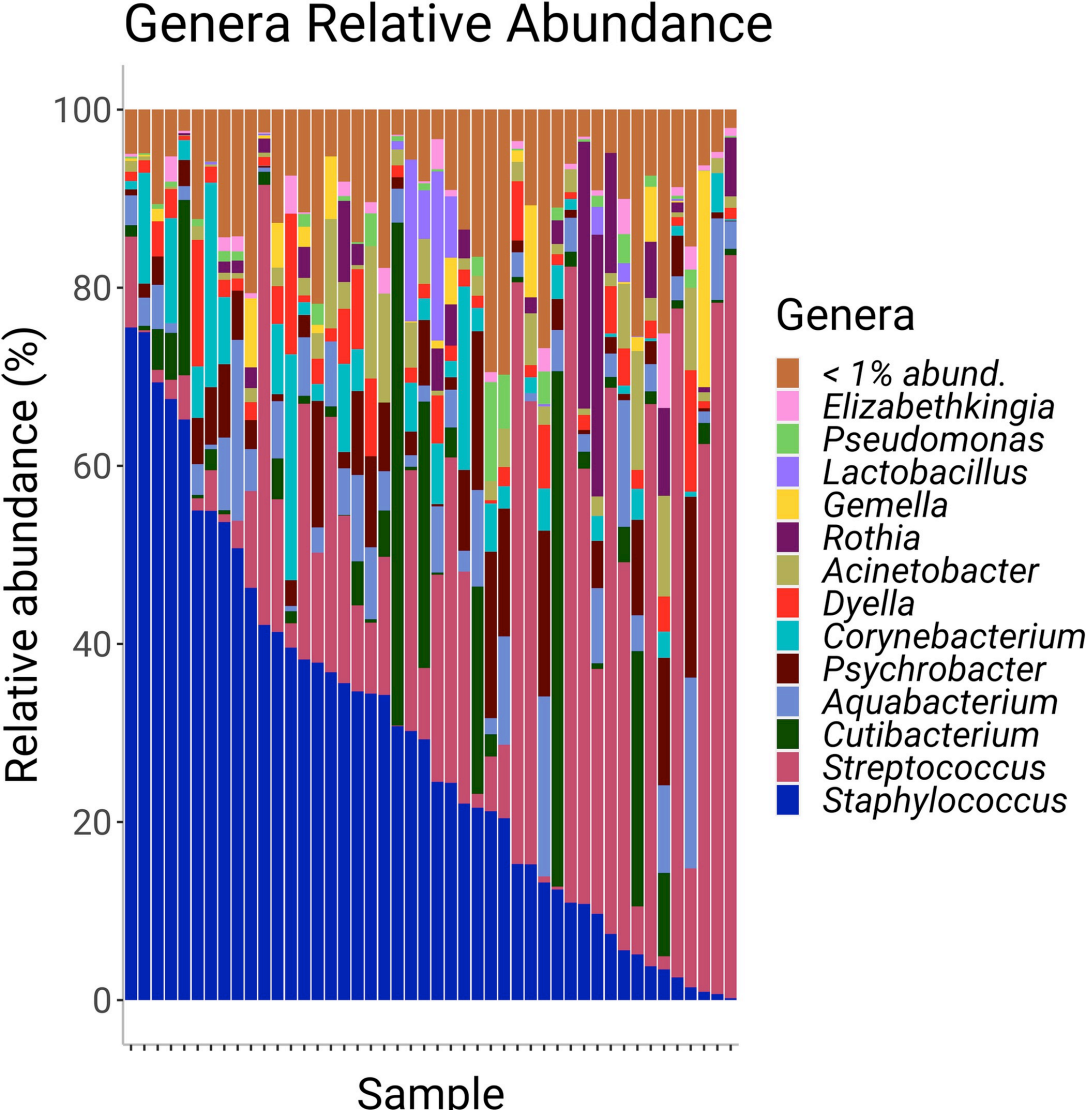
Relative abundance of bacterial genera within each participant’s milk. Samples are in descending order by relative abundance of *Staphylococcus*. The genera legend is in ascending order of relative abundance across all samples.

HMM genera with the highest relative abundance (Fig 1) were *Staphylococcus* with a mean ± s.d. relative abundance of 29 ± 22%, *Streptococcus* (24 ± 26%), *Cutibacterium* (6 ± 13%), *Aquabacterium* (6 ± 5%), *Psychrobacter* (5 ± 6%), *Corynebacterium* (4 ± 6%), *Dyella* (3 ± 4%), *Acinetobacter* (3 ± 4%), *Rothia* (3 ± 7%), *Gemella* (2 ± 4%), *Lactobacillus* (1 ± 4%), *Pseudomonas* (1 ± 2%), and *Elizabethkingia* (1 ± 2%). Relative abundance of HMM genera varied across individuals, with some genera that represented <1% across the entire sample population being present at >5% in milk produced by some individuals; these taxa including *Acidovorax*, *Candidatus thiophysa*, *Enhydrobacter*, Unclassified Neisseriaceae, *Janthinobacterium*, *Paeniclostridium*, *Polaromonas*, *Romboutsia*, and *Sphingomonas*.

### Associations of breastfeeding patterns with the human milk microbiome

#### Breastfeeding patterns and HMM α-diversity

**Fig 2:**
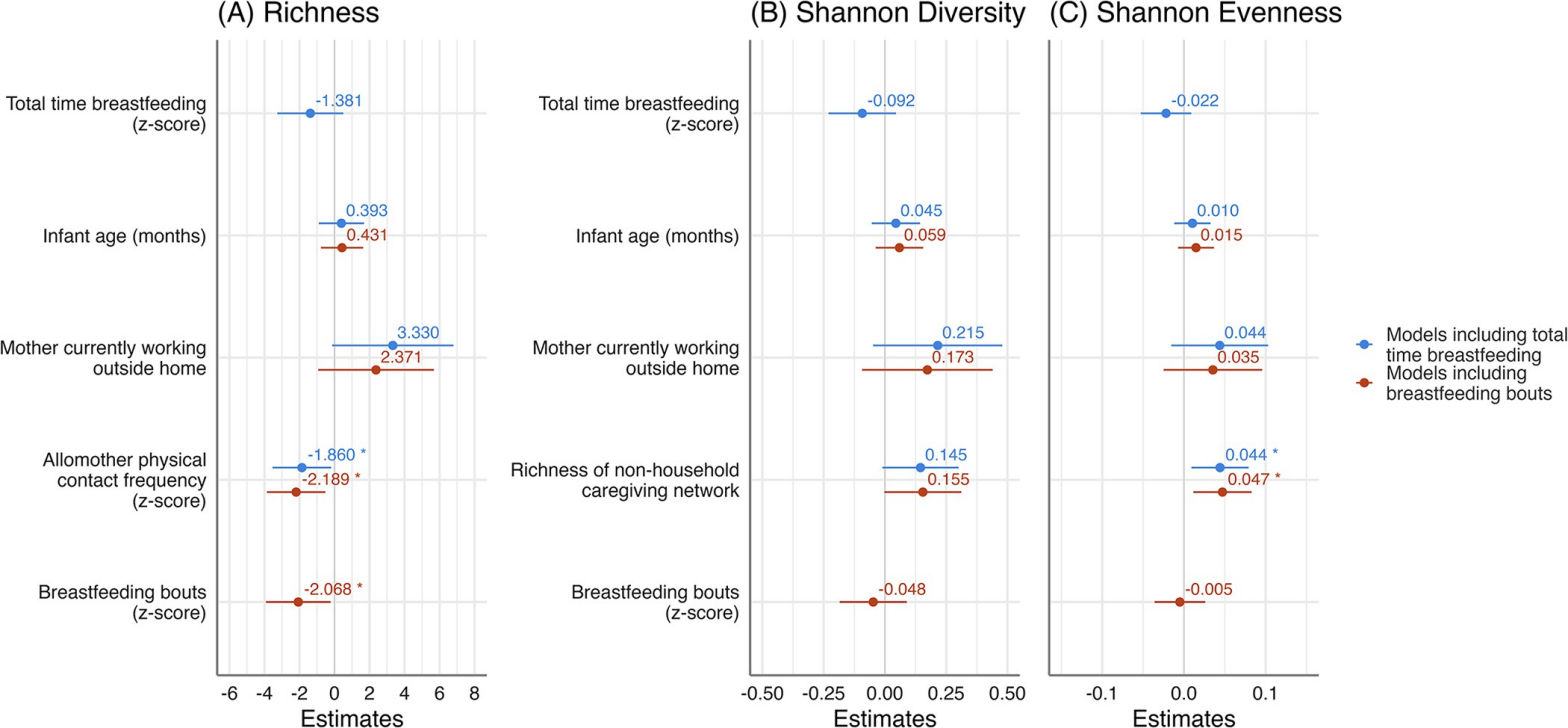
Regression models of α-diversity measures. Points are unstandardized *β* estimates. Lines are 95% confidence intervals. For models including total time breastfeeding as main predictor (blue); (A) predicting richness, adj. R^2^ = 0.09 (p = 0.09), (B) predicting Shannon diversity, adj. R^2^ = 0.12 (p = 0.06), and (C) predicting Shannon evenness, adj. R^2^ = 0.18 (p = 0.02). For models including breastfeeding bouts as main predictor (red); (A) predicting richness, adj. R^2^ = 0.15 (p = 0.03), (B) predicting Shannon diversity, adj. R^2^ = 0.09 (p = 0.10), and (C) predicting Shannon evenness, adj. R^2^ = 0.14 (p = 0.04). *p<0.05.

There were associations between number of breastfeeding bouts and HMM richness, but not with any other measures of HMM α-diversity. In the linear regression models predicting HMM richness including relevant covariates [Fig 2(A) model including total time breastfeeding in blue, adj. R^2^ = 0.09, p = 0.09; Fig 2(A) model including breastfeeding bouts in red, adj. R^2^ = 0.15, p = 0.03], HMM richness was negatively associated with frequency of breastfeeding bouts and allomother physical contact frequency. Specifically, frequency of breastfeeding bouts was inversely related to HMM richness (*β* = -2.07, p = 0.03). Allomother physical contact frequency was also inversely related to HMM richness (*β* = -1.86, p = 0.03 in model including total time breastfeeding and *β* = -2.19, p = 0.01 in model including breastfeeding bouts). There was a trend toward maternal work outside the home being positively associated with HMM richness in the model that included total time breastfeeding (*β* = 3.33, p = 0.06).

Shannon diversity [Fig 2(B) model including total time breastfeeding in blue, adj. R^2^ = 0.12, p = 0.06; Fig 2(B) model including breastfeeding bouts in red, adj. R^2^ = 0.09, p = 0.10] was not independently associated with any variable. However, non-household caregiving network trended positively with Shannon diversity in the model that included breastfeeding bouts (*β* = 0.15, p = 0.05), and in the model that included total time breastfeeding (*β* = 0.15, p = 0.07). Shannon evenness [Fig 2(C) model including total time breastfeeding in red, adj. R^2^ = 0.18, p = 0.02; Fig 2(C) model including breastfeeding bouts in blue, adj. R^2^ = 0.14, p = 0.04] was positively associated with non-household caregiving network in the model including total time breastfeeding (*β* = 0.04, p = 0.01) and the model including breastfeeding bouts (*β* = 0.05, p = 0.01).

#### Breastfeeding patterns and differential abundance of bacterial genera in the HMM

**Fig 3:**
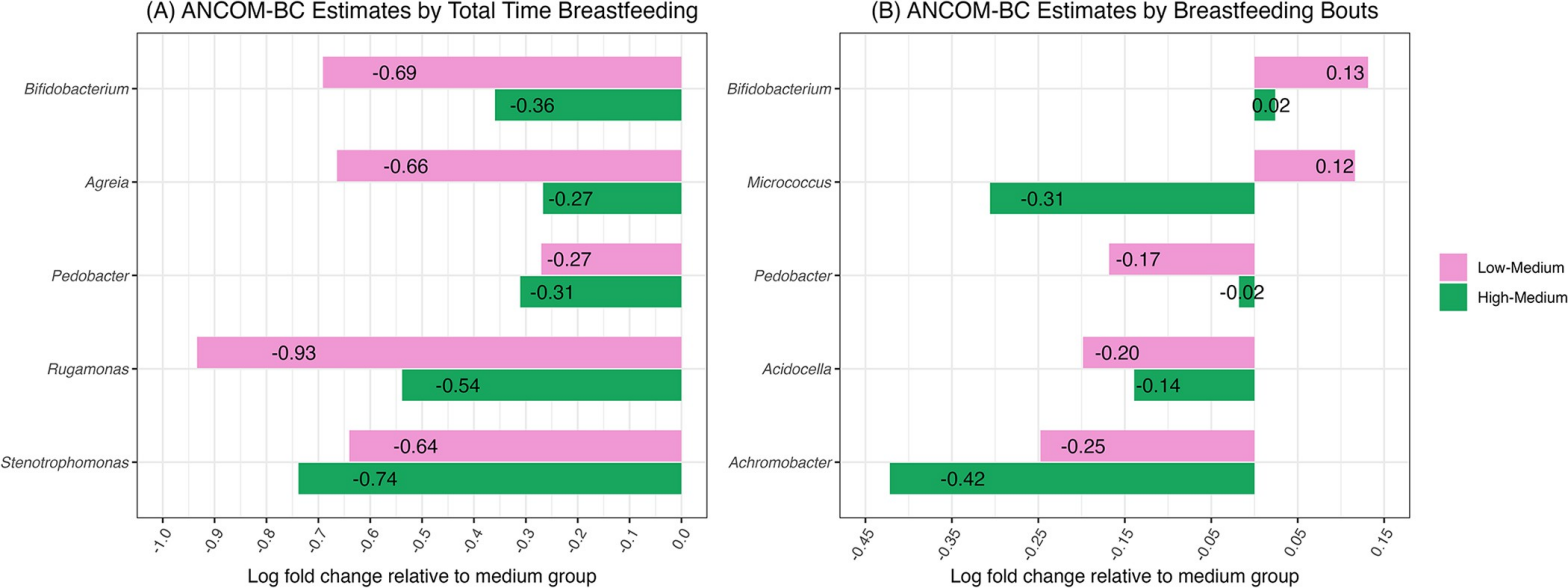
Log-fold change estimates relative to medium group, of genera identified as differentially abundant across (A) total time breastfeeding groups and (B) breastfeeding bouts groups in ANCOM-BC global test. Low <-1 z-score, medium [–1, 1] z-score, high >1 z-score. Genera indicated in plots had global test q<0.05. Genera are ordered phylogenetically.

There were significant differences in the estimated absolute abundance of several bacterial genera by breastfeeding patterns. Across total time breastfeeding groups, of the 35 genera evaluated in the ANCOM-BC, 5 were differentially abundant (Fig 3), including *Bifidobacterium*, *Agreia*, *Pedobacter*, *Rugamonas*, and *Stenotrophomonas*. Across breastfeeding bouts groups, 5 of 35 genera were differentially abundant, specifically *Bifidobacterium*, *Micrococcus*, *Pedobacter*, *Acidocella*, and *Achromobacter*. Differential abundance of genera did not significantly vary by any other covariate besides the main predictor variables (total time breastfeeding and breastfeeding bouts groups), although in the total time breastfeeding model, infant age trended (β = -0.42, q = 0.06) with abundance of S*taphylococcus* and frequency of allomother physical contact trended (β = 0.005, q = 0.06) with *Dyella*. In the breastfeeding bouts model, infant age trended (β = -0.27, q = 0.09) with *Streptomyces* and *Staphylococcus* (β = -0.37, q = 0.09), and frequency of allomother physical contact trended (β = 0.004, q = 0.06) with *Acidocella*. No other covariates were associated with other genera at q<0.10.

However, the directions of these differences were not consistent across the two ANCOM-BC analyses, based on the log-fold change estimates of the abundance of genera for low and high total time breastfeeding and breastfeeding bouts groups, compared to the reference (medium) group (Fig 3). Only *Bifidobacterium* and *Pedobacter* were significantly differentially abundant across both breastfeeding bouts and total time breastfeeding groups. *Pedobacter* log-fold change estimates reflected an inverted U-shape, i.e., lower estimates for both the low and high groups relative to the medium group, for both total time breastfeeding and breastfeeding bouts groups (e.g., an estimated -0.27 abundance in the low total time breastfeeding group compared to the medium group, and an estimated -0.31 abundance in the high total time breastfeeding group compared to the medium group). *Bifidobacterium* estimates were opposite between the models, with an inverted U-shape between the total time breastfeeding groups (lower abundances estimated in the low and high groups relative to the medium group) and a U-shape between the breastfeeding bouts groups (higher abundances estimated in the low and high groups relative to the medium group). *Agreia*, *Rugamonas*, and *Stenotrophomonas* estimates reflected an inverted U-shape across total time breastfeeding groups, with lower abundances estimated in the low and high groups relative to the medium group. *Acidocella* and *Achromobacter* estimates showed an inverted U-shape for breastfeeding bouts groups, with lower abundances estimated in the low and high groups relative to the medium group. *Micrococcus* estimates decreased from low to high breastfeeding bouts groups, with the low group showing a 0.12 log-fold change over the medium group, and the high group showing a decrease of -0.31 log-fold change compared to the medium group.

## Discussion

### HMM composition compared to other populations

The most relatively abundant genera (e.g., *Streptococcus* and *Staphylococcus*) in this study were similar to those reported previously [11,46–49]. While there is a wide variety of bacterial genera in the HMM across populations and individuals, there appears to be a core bacterial profile. Others have reported that the HMM is mostly comprised of gram-positive Bacillota, *Streptococcus* and *Staphylococcus* [9,12,14,50], which was also the case in the present study. Actinomycetota such as *Corynebacterium* are also frequently found in high abundance [48,50,51], as was the case in the current study. Conversely, multiple genera often predominant in human milk were not highly abundant in the present study, including *Bifidobacterium*, *Propionibacterium*, as well as gram-negative Pseudomonadota *Ralstonia*, *Sphingomonas*, and *Bradyrhizobium* [48,50,51].

HMM richness in this study was somewhat lower compared to some other published studies, such as an average richness of 147 amplicon sequence variants (ASVs) in milk collected from 393 Canadian women [18] and a median richness of 376 operational taxonomic units (OTUs) in milk collected from 67 women in Mexico [52], although this variability may be attributable to methodological differences in sampling and analysis such as rarefying to a higher number of reads ([18] rarefied to 25,000). Shannon diversity was somewhat lower than that reported previously in milk produced by Mexican and Irish women [52,53]. In summary, the microbial communities and diversity found in milk collected for this study were generally within the variation reported by other studies, although some differences are noted.

### Breastfeeding patterns and HMM α-diversity

Our data support our hypothesis that breastfeeding patterns are associated with HMM α-diversity, specifically richness, when accounting for other mother-infant ecological factors. The number of breastfeeding bouts was inversely associated with HMM richness (an estimated two fewer ASVs present for each standard deviation increase in breastfeeding bouts), when accounting for covariates. However, total time breastfeeding was not associated with any HMM α-diversity measure after accounting for covariates. HMM Shannon diversity and evenness were also not associated with breastfeeding patterns. This demonstrates a relationship between the incidence of unique bacteria taxonomic units in HMM and breastfeeding patterns, rather than the abundance or distribution of bacteria taxa in HMM.

In general, lower α-diversity in HMM has been associated with adverse maternal health conditions, including obesity and gestational pre-hypertension [11,54]. However, the reduced bacterial richness in milk associated with more breastfeeding bouts may indeed point to a calibration of a shared mother-infant microbiome. Lower infant GI microbiome diversity among exclusively breastfed infants transitioning to solid foods may reflect a highly adaptable, flexible GI microbiome in early life to calibrate to the particular environment [23]. Miller [55] proposed that this reduced infant GI microbiome diversity–or a more flexible, adaptable microbiome–may be a type of developmental constraint (limits on possible phenotypic expression) resulting from maternal immunological signals conveyed through milk. Considerable research is needed to determine the concordance of the infant GI microbiome with the maternal milk microbiome given the frequency and duration of breastfeeding.

These results also indicate that it is important to account for both frequency and patterns of breastfeeding in HMM research. Total time breastfeeding was not associated with any measure of HMM α-diversity after accounting for selected covariates, although it trended negatively with all measures. However, number of breastfeeding bouts was negatively associated with richness, but not with the abundance-related indices of Shannon diversity and evenness. These findings indicate that different aspects of breastfeeding behavior may be important for shaping different measures of HMM α-diversity. For instance, more frequent breastfeeding is associated with lower average milk volume per feed [56], meaning that some milk may remain in the mammary gland for a longer period. This may influence which bacteria can become established, reflected in richness measures, but not necessarily abundance-related measures such as Shannon diversity and evenness.

### Infant caregiving ecology and HMM α-diversity

We also found associations between the infant caregiving ecology and HMM α-diversity. Allomother physical contact with the infant was associated with decreased HMM richness (an estimated two fewer ASVs present for each standard deviation increase in allomother physical contact frequency), and richness of the non-household caregiving network was positively associated with HMM evenness, when accounting for covariates. Presumably, more frequent allomother physical contact with the infant would introduce more types of bacteria to the infant, and under the infant seeding hypothesis of the HMM would lead to increased HMM α-diversity. However, this is contrary to our results showing decreased HMM richness with more allomother physical contact. Rather than reflecting infant bacterial exposures exclusive from the mother, the frequency of allomother physical contact may reflect a difference in maternal-infant social ecology and caregiving practices that determine the broader microbial environment in which mother-infant dyads live [9,14]. It is particularly notable that frequency of breastfeeding bouts and infant caregiving ecology (allomother physical contact frequency and richness of the non-household caregiving network) were predictors of HMM α-diversity measures while selected covariates (e.g., infant age) were not.

These results may also indicate important social ecological influences on the HMM. In a study of HMM composition among hunter-gatherer and horticulturalist mothers and infants in the Central African Republic (CAR), we reported that more intensive allomother caregiving was associated with greater HMM diversity and evenness but was not associated with richness. We found some similarities here in that Shannon evenness was associated with the non-household caregiving network. However, we also found that the frequency of allomother physical contact was negatively associated with HMM richness. These differences likely reflect variability in the infant caregiving ecology between hunter-gatherers and horticulturalists in the CAR and the US population in the current study. In CAR, circumstances including intensive maternal caregiving and more frequent maternal breastfeeding would be when mothers bring their infants with them on trips, exposing the infant and mother to a wider range of microbial environments. In the US, however, more intensive maternal caregiving is likely to reflect more maternal-infant time at home, reduced exposure to others, and a smaller range of microbial environments. This ecological impact on HMM diversity and composition is reflected in decreased richness with more breastfeeding bouts, possibly indicating increased maternal-infant time at home. The frequency of allomother physical contact with the infant is also a predominantly household exposure measure, with most individuals touching the infant also being household members. The present study found that the more different types of people from outside the household who are in contact with the infant, the greater the HMM evenness.

### Breastfeeding patterns and differential abundance of bacterial genera in HMM

Multiple genera were differentially abundant across breastfeeding pattern groups. Notably, none of these genera were present with an average relative abundance of >1%. As the ANCOM-BC analysis included structural zeroes between groups in calculating its global test of differential abundance, this indicates that many of these differences across groups can be attributed to the presence vs. absence of the genus between the groups. This comports with the models of the α-diversity indices, demonstrating more associations with richness (presence/absence of different taxa) as opposed to evenness and Shannon diversity which further account for taxa abundance. Overall, these results indicate that breastfeeding patterns are associated with differential abundance of low-abundant genera in varied directions, after accounting for covariates of infant age, maternal work outside the home, and allomother physical contact frequency.

Some of the genera that were differentially abundant between breastfeeding pattern groups have previously been found to differ based on environmental and behavioral factors. For instance, *Bifidobacteria* (which was estimated to be lowest abundance in the low total time breastfeeding group) was reported to be lower in milk produced by women who pump milk compared to those who only feed at the breast [18], and is differentially abundant between populations that vary in subsistence strategies [14,57]. *Micrococcus* was one of the top 10 most abundant genera present among women in the CAR [14], although not among a sample of US women [13], and was present at a low overall relative abundance in milk produced by women in the present study. In the only other study of breastfeeding patterns and HMM variation, more frequent breastfeeding was associated with decreased incidence of *Corynebacterium* and *Staphylococcus* [34]. We did not find differences in the abundance of those genera, possibly due to differences in study design (LeMay-Nedjelski and colleagues’ study did not collect milk aseptically, so the skin microbiome may also be present in milk samples) and differences in statistical analyses (regressions on the most abundant genera vs. ANCOM-BC across all genera).

### Strengths and limitations

Limitations to this study necessarily constrain generalization of these results. As this was a convenience sample, this is not representative of the population of breastfeeding mothers in the region or within the United States. Additionally, rarefying to 200 reads may have reduced the diversity measures and thus, the richness values are lower than those reported in other studies. Without infant oral microbiome data, we could not investigate whether the observed relationships might be due to infant oral microbiota “retrograde” backflow while breastfeeding. There are also potential biases introduced when using primers targeting various hypervariable regions of the 16S rRNA gene. Numerous studies have shown that choice of 16S rRNA region can affect estimates of taxonomic diversity [58–60]. So, consideration of the primers used is warranted when comparing our results to others who might have used primers targeting differing regions or the full length of the 16S rRNA gene or to those who conducted shotgun sequencing. There were also notable strengths of this study. Direct observations over the course of three days allowed for a more accurate, precise assessment of the infant caregiving ecology and actual breastfeeding behavior and patterns than the type of data typically obtained in this regard. Furthermore, milk samples were collected after the observations, establishing the necessary temporal relationship of exposure and outcome for this research question. Additionally, this analysis was able to consider many common covariates and predictors of the human milk microbiome in order to adequately identify the best predictors of HMM variation in this sample.

## Conclusions

In this cohort of exclusively breastfeeding US mothers and their infants, more frequent breastfeeding bouts were associated with decreased HMM richness, and both number of breastfeeding bouts and total time breastfeeding were associated with differential abundance of some low-abundant genera. Frequency of allomother physical contact with the infant was also negatively associated with HMM richness. Infant non-household caregiving network was positively associated with HMM evenness. These results point to the potential impact of breastfeeding patterns on the bacterial diversity and community composition of human milk, as well as the potential impacts of different aspects of breastfeeding behaviors (total time vs. frequency of bouts). Overall, these results provide support for the mother-infant nexus framework. HMM variation is associated with the dynamic interplay of the mother-infant dyad via breastfeeding [25]. Future research should identify whether these patterns are observed in other populations and whether these patterns are consistent with infant GI microbiome diversity measures. Importantly, future research needs to clarify the biological mechanisms whereby breastfeeding bouts can shape HMM composition and diversity.

## Supporting information

S1 Fig Breastfeeding patterns across participants (r = 0.60, p<0.001). (DOCX)

S2 Fig Distribution of covariates, demographic variables, and HMM α-diversity measures across groups. (DOCX)

S3 Fig Correlation matrix of all model covariates. Pearson correlation coefficients. *p-value <0.05, **p-value <0.01, ***p-value <0.001.
(DOCX)

S4 Fig Relative abundance of all genera within breastfeeding bouts and total time breastfeeding groups. (DOCX)

S1 Table Regression models with considered covariates compared to final models. Each column is a single model, with the first row displaying the model fit statistics (Adj. R^2^, residual standard error, model p-value), and the following rows displaying the coefficient values for each listed independent variable.
(DOCX)
