# Supplementary figures and images for: Breastfeeding patterns are associated with human milk microbiome composition: The Mother-Infant Microbiomes, Behavior, and Ecology Study (MIMBES)
Source: PLoS One. 2023 Aug 9;18(8):e0287839. doi: 10.1371/journal.pone.0287839 (PMC10411759; doi:10.1371/journal.pone.0287839)

**
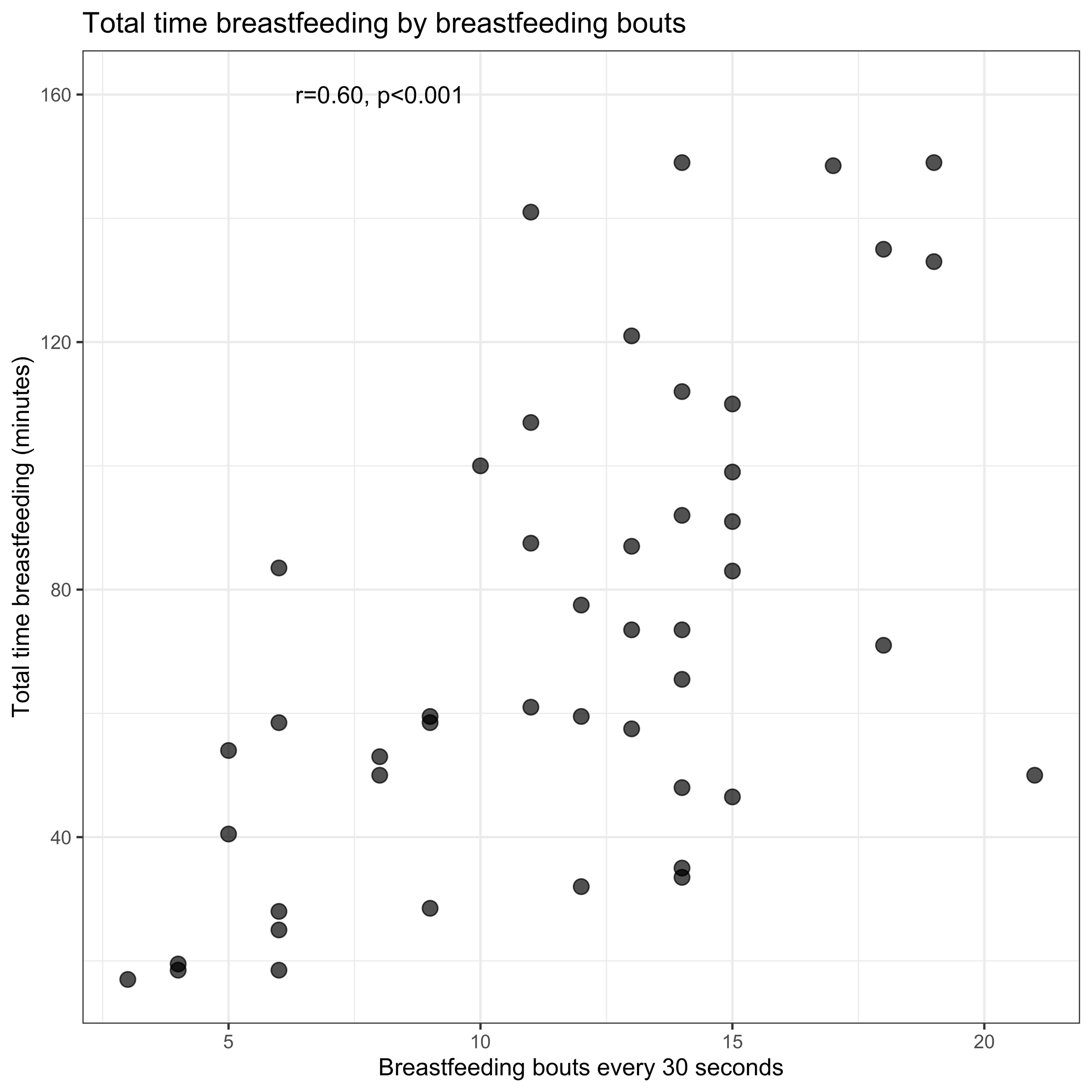
**

Supplement: S1 Fig — (DOCX) [file pone.0287839.s001.docx]

**Figure A**

**
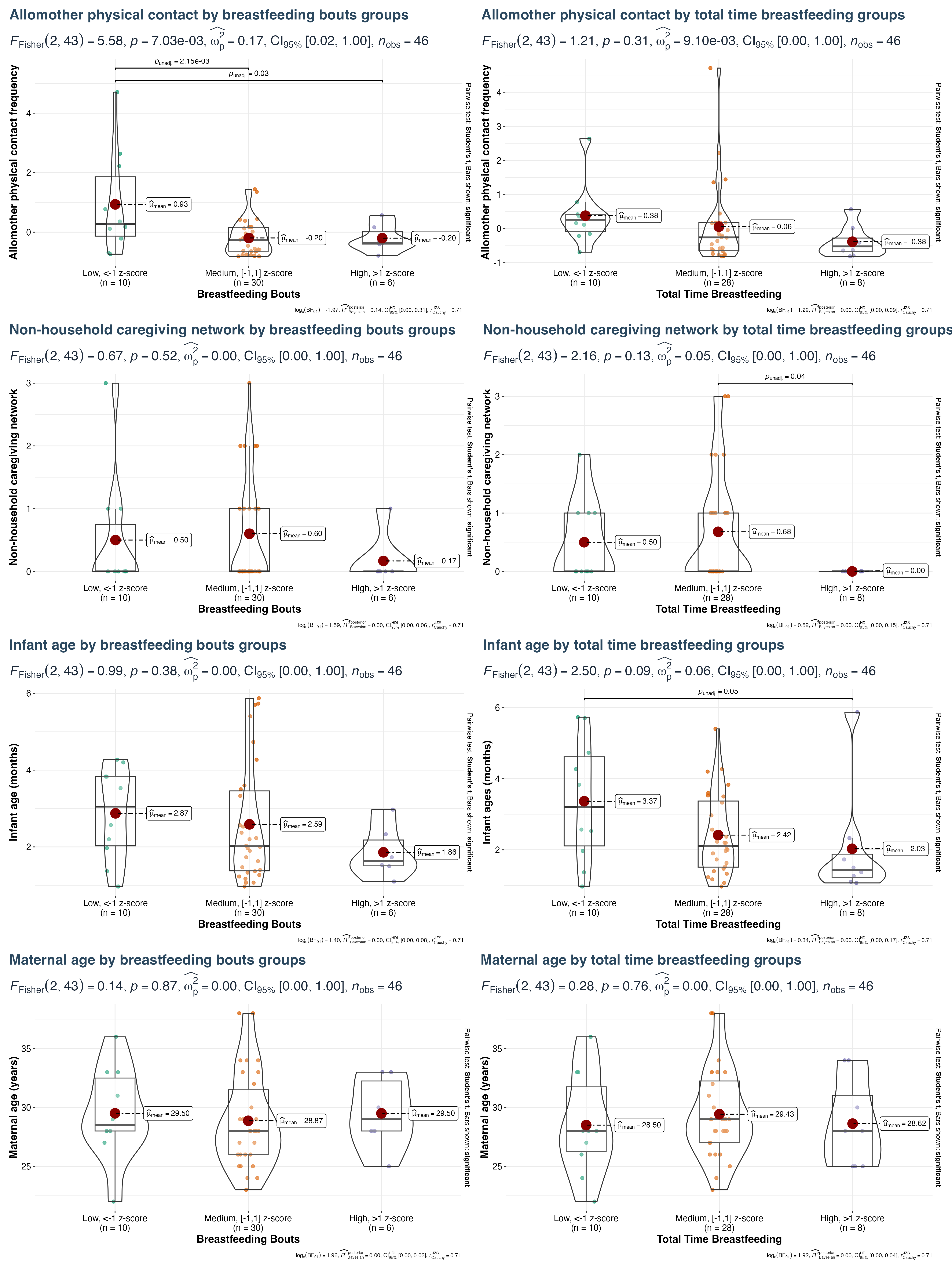
**

**Figure B**


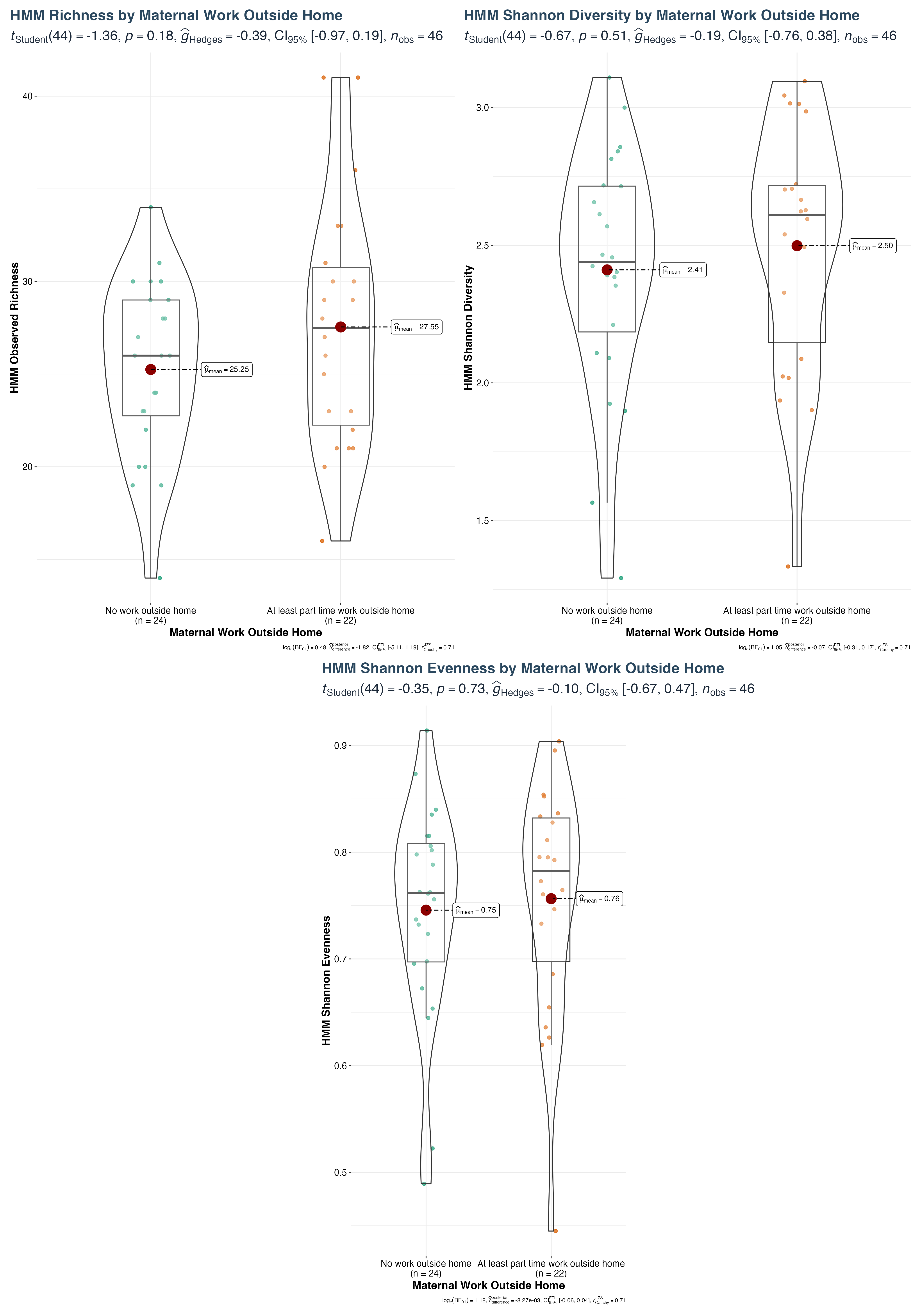


**Figure C**


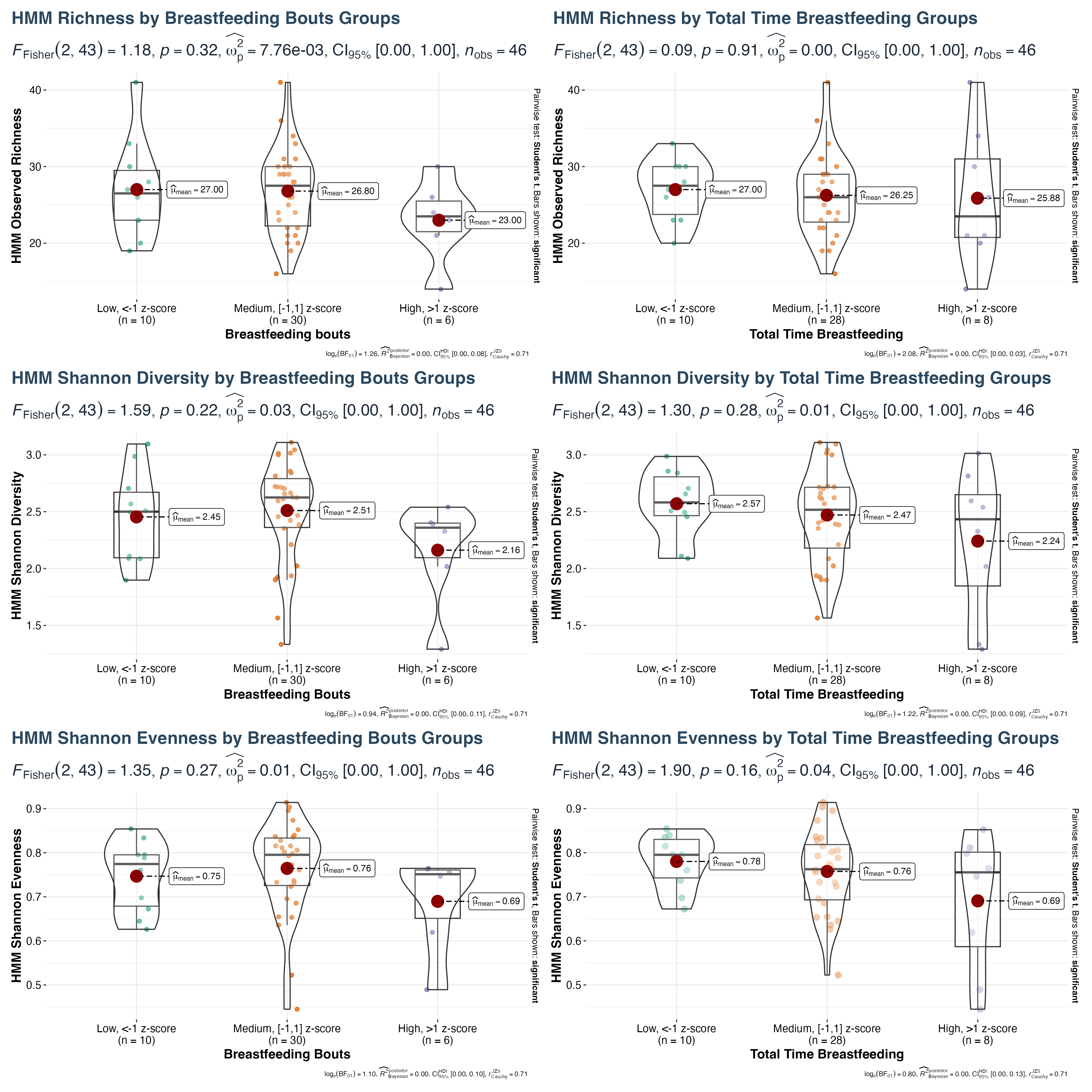

Supplement: S2 Fig — (DOCX) [file pone.0287839.s002.docx]

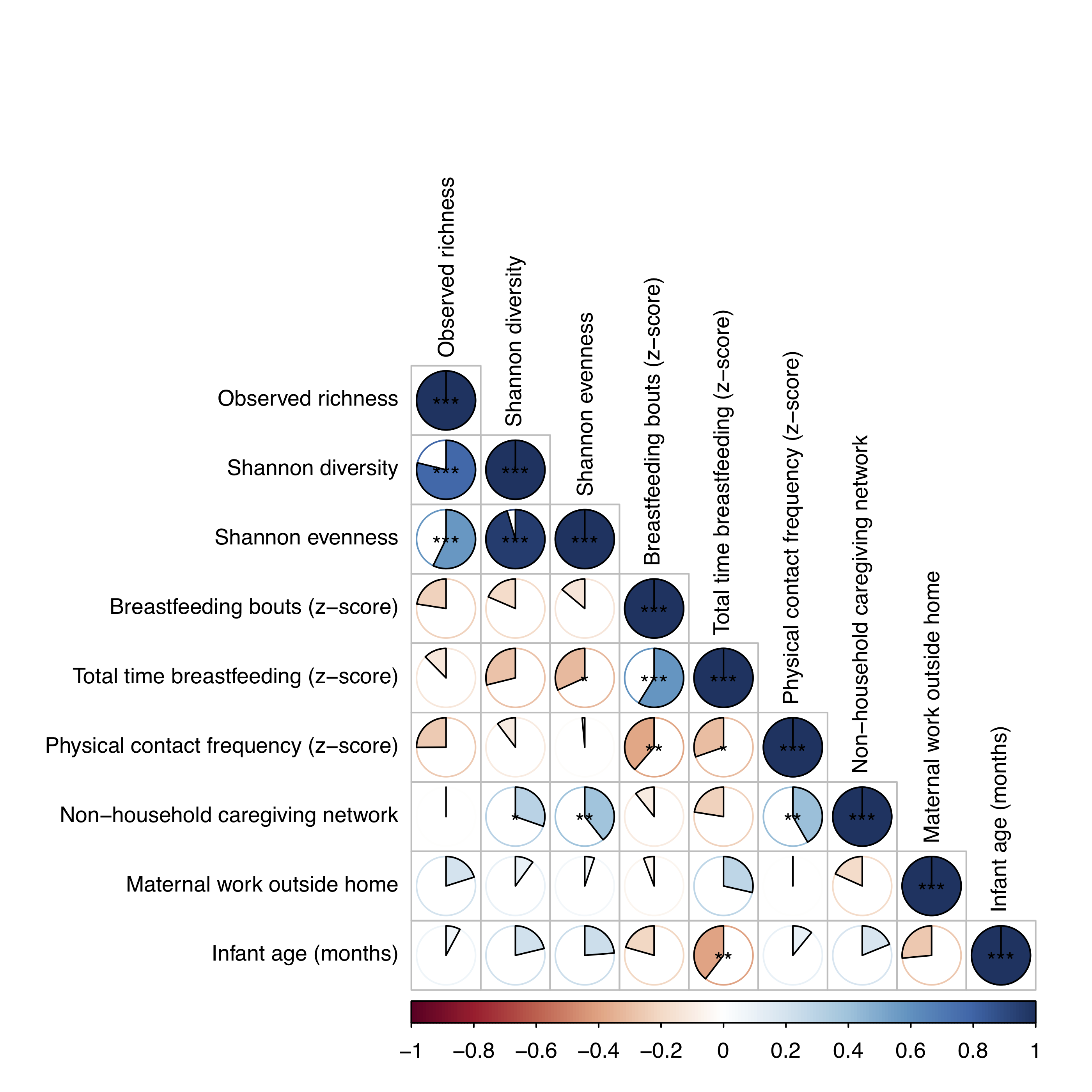

Supplement: S3 Fig — Pearson correlation coefficients. *p-value <0.05, **p-value <0.01, ***p-value <0.001. (DOCX) [file pone.0287839.s003.docx]

**Figure A**


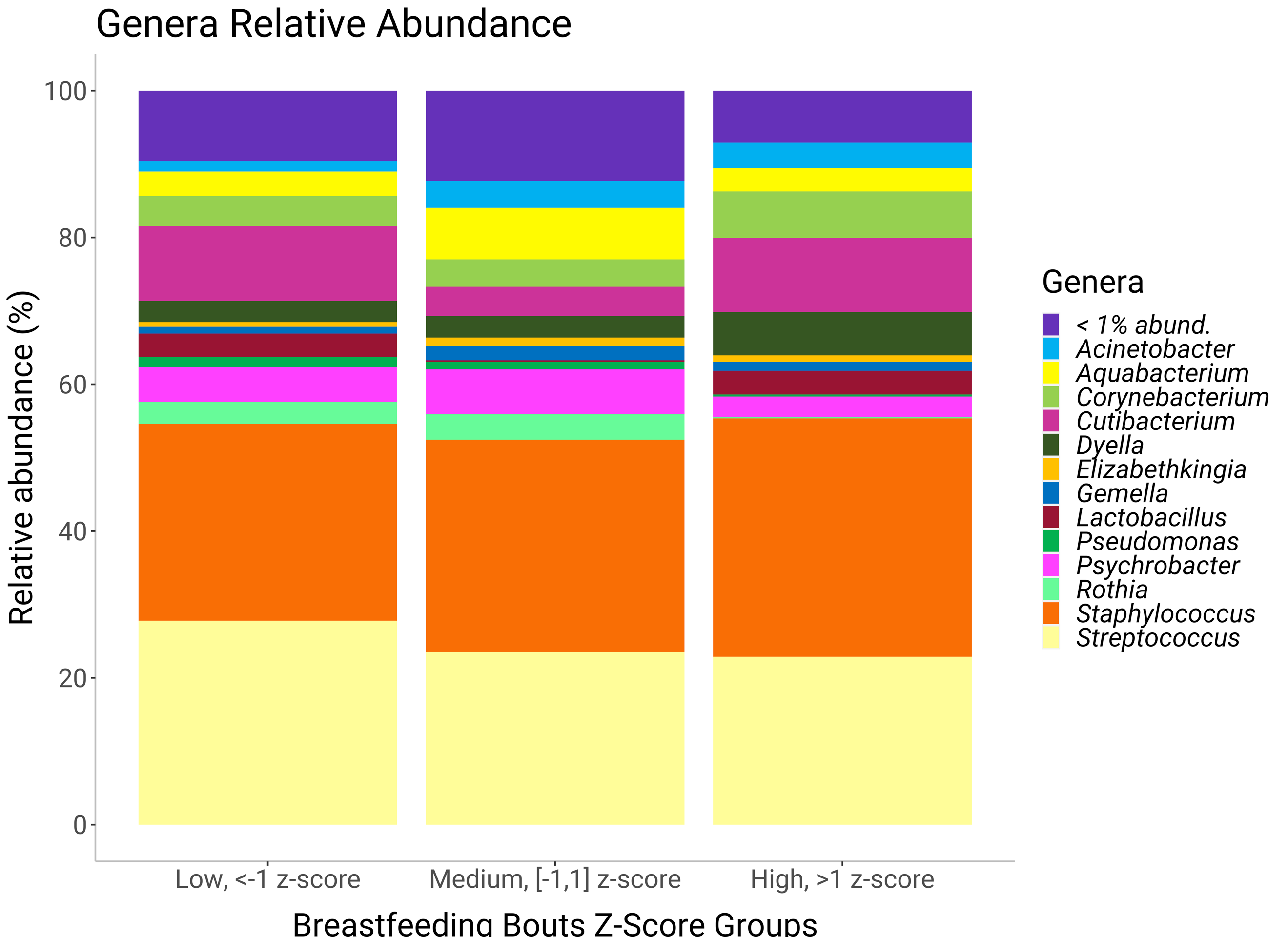


**Figure B**


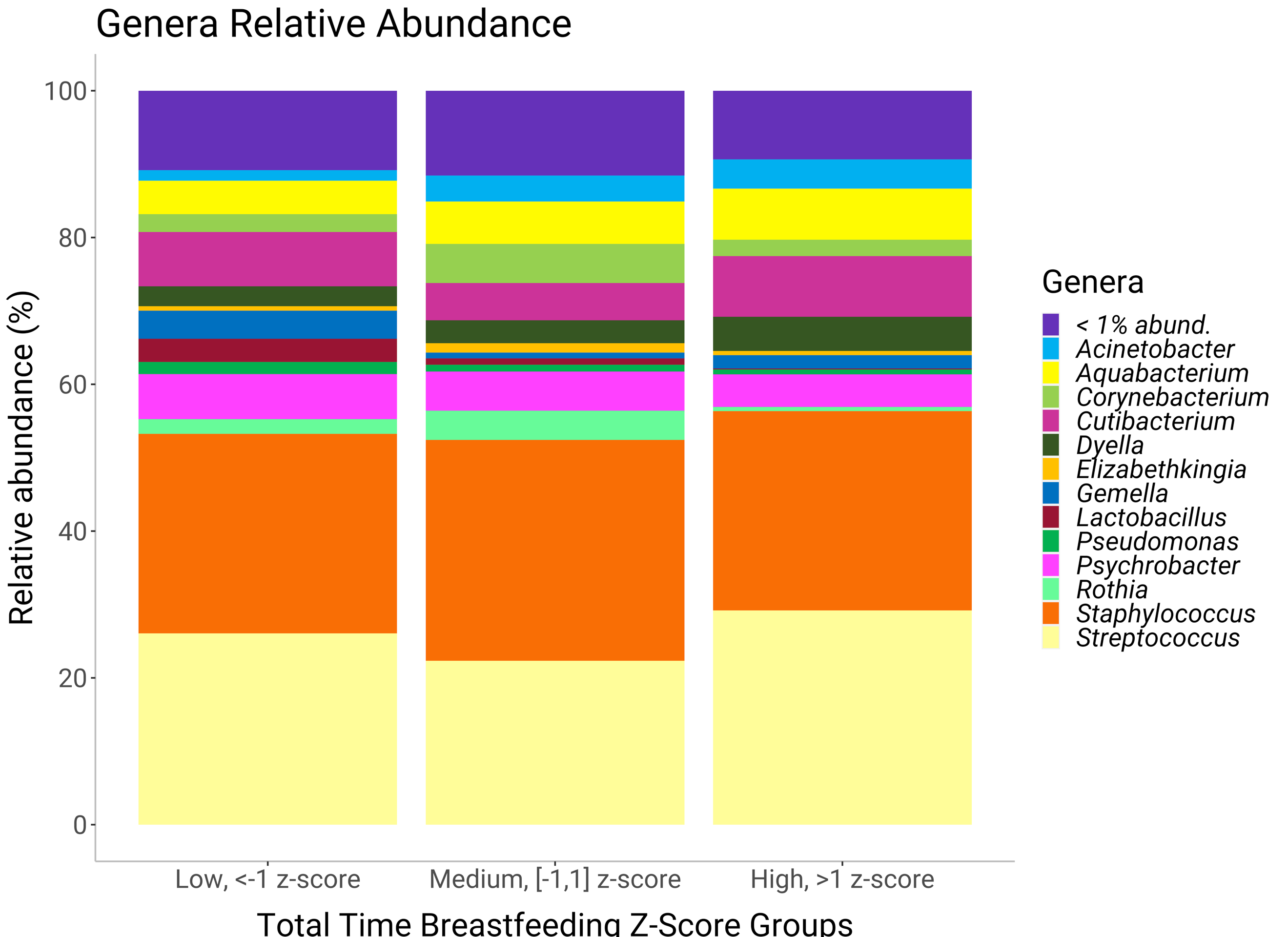

Supplement: S4 Fig — (DOCX) [file pone.0287839.s004.docx]
