# Supplementary material for: Breastfeeding patterns are associated with human milk microbiome composition: The Mother-Infant Microbiomes, Behavior, and Ecology Study (MIMBES)
Source: PLoS One. 2023 Aug 9;18(8):e0287839. doi: 10.1371/journal.pone.0287839 (PMC10411759; doi:10.1371/journal.pone.0287839)
Supplement: S1 Table — Each column is a single model, with the first row displaying the model fit statistics (Adj. R2, residual standard error, model p-value), and the following rows displaying the coefficient values for each listed independent variable. (DOCX) [file pone.0287839.s005.docx]

**Table A**

|  | **Breastfeeding bouts** | | **Total time breastfeeding** | |
| --- | --- | --- | --- | --- |
|  | **Richness final model** | Richness considered model | **Richness final model** | Richness considered model |
| Model adj. R^2^, residual standard error (p-value) | 0.15, 5.31 (0.03) | 0.01, 5.74 (0.38) | 0.09, 5.48 (0.09) | -0.02, 5.81 (0.52) |
| Coefficient β ± SE  (p-value, 95% CI) | | | | |
| Total time breastfeeding (z-score) |  |  | -1.38 ± 0.93  (0.15, -3.27:0.50) | -0.76 ± 0.95  (0.43, -2.68:1.17) |
| Infant age (months) | 0.43 ± 0.60  (0.48, -0.78:1.64) | 0.39 ± 0.65  (0.55, -0.92:1.71) | 0.39 ± 0.64  (0.54, -0.90:1.69) | 0.39 ± 0.68  (0.57, -0.98:1.77) |
| Maternal work outside the home | 2.37 ± 1.64  (0.15, -0.93:5.68) | 2.45 ± 1.79  (0.18, -1.17:6.06) | 3.33 ± 1.72  (0.06, -0.13:6.79) | 2.99 ± 1.82  (0.11, -0.69:6.67) |
| Allomother physical contact frequency (z-score) | -2.19 ± 0.83  (0.01, -3.86:-0.52) |  | -1.86 ± 0.83  (0.03, -3.54:-0.18) |  |
| Non-household caregiving network |  | -0.04 ± 1.06  (0.97, -2.18:2.09) |  | -0.04 ± 1.08  (0.97, -2.21:2.13) |
| Breastfeeding bouts (z-score) | -2.07 ± 0.92  (0.03, -3.92:-0.22) | -1.16 ± 0.92  (0.21, -3.03:0.70) |  |  |

**Table B**

|  | **Breastfeeding bouts** | | **Total time breastfeeding** | |
| --- | --- | --- | --- | --- |
|  | **Shannon diversity final model** | Shannon diversity considered model | **Shannon diversity final model** | Shannon diversity considered model |
| Model adj. R^2^, residual standard error (p-value) | 0.09, 0.42 (0.10) | 0.04, 0.43 (0.23) | 0.12, 0.42 (0.06) | 0.09, 0.42 (0.10) |
| Coefficient β ± SE  (p-value, 95% CI) | | | | |
| Total time breastfeeding (z-score) |  |  | -0.09 ± 0.07  (0.18, -0.23:0.05) | -0.14 ± 0.07  (0.05, -0.29:0.003) |
| Infant age (months) | 0.06 ± 0.05  (0.23, -0.04:0.16) | 0.07 ± 0.05  (0.15, -0.03:0.17) | 0.04 ± 0.05  (0.37, -0.05:0.14) | 0.05 ± 0.05  (0.29, -0.05:0.15) |
| Maternal work outside the home | 0.17 ± 0.13  (0.20, -0.09:0.44) | 0.13 ± 0.13  (0.34, -0.14:0.40) | 0.22 ± 0.13  (0.11, -0.05:0.48) | 0.20 ± 0.13  (0.13, -0.06:0.47) |
| Allomother physical contact frequency (z-score) |  | -0.09 ± 0.07  (0.20, -0.23:0.05) |  | -0.09 ± 0.06  (0.15, -0.22:0.03) |
| Non-household caregiving network | 0.15 ± 0.08  (0.05, -0.00:0.31) |  | 0.15 ± 0.08  (0.07, -0.01:0.30) |  |
| Breastfeeding bouts (z-score) | -0.05 ± 0.07  (0.49, -0.18:0.09) | -0.10 ± 0.07  (0.20, -0.25:0.05) |  |  |

**Table C**

|  | **Breastfeeding bouts** | | **Total time breastfeeding** | |
| --- | --- | --- | --- | --- |
|  | **Shannon evenness final model** | Shannon evenness considered model | **Shannon evenness final model** | Shannon evenness considered model |
| Model adj. R^2^, residual standard error (p-value) | 0.14, 0.10 (0.04) | -0.01, 0.10 (0.46) | 0.18, 0.09 (0.02) | 0.06, 0.10 (0.16) |
| Coefficient β ± SE  (p-value, 95% CI) | | | | |
| Total time breastfeeding (z-score) |  |  | -0.02 ± 0.02  (0.16, -0.05:0.01) | -0.03 ± 0.02  (0.07,  -0.07:0.003) |
| Infant age (months) | 0.01 ± 0.01  (0.18, -0.01:0.04) | 0.02 ± 0.01  (0.12, -0.01:0.04) | 0.01 ± 0.01  (0.35, -0.01:0.03) | 0.01 ± 0.01  (0.27, -0.01:0.04) |
| Maternal work outside the home | 0.04 ± 0.03  (0.24, -0.02:0.10) | 0.02 ± 0.03  (0.47, -0.04:0.09) | 0.04 ± 0.03  (0.14, -0.02:0.10) | 0.04 ± 0.03  (0.24, -0.03:0.10) |
| Allomother physical contact frequency (z-score) |  | -0.01 ± 0.02  (0.60, -0.04:0.02) |  | -0.01 ± 0.02  (0.40, -0.04:0.02) |
| Non-household caregiving network | 0.05 ± 0.02  (0.01, 0.01:0.08) |  | 0.04 ± 0.02  (0.01, 0.01:0.08) |  |
| Breastfeeding bouts (z-score) | -0.005 ± 0.02  (0.75, -0.04:0.03) | -0.01 ± 0.02  (0.50, -0.05:0.02) |  |  |
